# Hemorrhagic cholecystitis with rare imaging presentation: a case report and a lesson learned from neglected medication history of NSAIDs

**DOI:** 10.1186/s12876-020-01312-0

**Published:** 2020-06-05

**Authors:** Xin Zhang, Chunjun Zhang, Haifeng Huang, Junsen Wang, Yun Zhang, Qida Hu

**Affiliations:** 1 Department of Radiology, First Affiliated Hospital, Zhejiang University School of Medicine, Hangzhou, 310003 China; 2 Department of Surgery, Shengzhou People’s Hospital, Shengzhou, 312400 China; 3 Department of Pathology, Shengzhou People’s Hospital, Shengzhou, 312400 China; 4 Department of Hepatobiliary and Pancreatic Surgery, First Affiliated Hospital, Zhejiang University School of Medicine, 79 Qingchun Road, Hangzhou, 310003 China

**Keywords:** Hemorrhagic cholecystitis, Gallbladder cancer, Medication history, NSAIDs abuse, Case report

## Abstract

**Background:**

Gallbladder carcinogenesis, frequently occurredin chronic cholecystitis patients, requires radical resection. We herein describe a hemorrhagic cholecystitis case that failed to be differentiated from gallbladder cancer preoperatively owing to the neglected medication history of long term oral nonsteroidal anti-inflammatory drugs (NSIADs) intake.

**Case presentation:**

A 57-year-old Chinese female was admitted for right upper quadrant pain with the initial diagnosis of cholecystitis. Radiological studies were unable to exclude the differential diagnosis of suspected gallbladder cancer. During the scheduled radical resection of the suspected lesions, the gross dissection showed an interesting presentation of hemorrhagic cholecystitis, without any pathological evidence of malignancies. Additional postoperative investigation revealed a neglected medication history of long-term NSAIDs use.

**Conclusions:**

This case suggests the importance of preoperative review of medication history and patient education on prescription drug abuse.

## Background

Gallstone and its related cholecystitis are commonly found in Southeastern Asia, with an estimated prevalence rate of 5–20% [1]. Long term gallstone-associated cholecystitis may increase the risk of gallbladder carcinoma [2, 3], a highly malignant tumor with poor prognosis [4]. However, the differential diagnosis between cholecystitis and gallbladder cancer could remain unclear during the preoperative stage, as malignancies are frequently revealed intraoperatively [5, 6]. It is essential to determine the nature of the disease before the end of the cholecystectomy to avoid neglecting potential carcinogenesis lesions. At the same time, owing to the surgical principle of negative margin, any lesion in suspicious of malignant presentation will be resected radically with wedge hepatectomy, even if it is a false-positive [7, 8]. Therefore, ruling out malignancies with clear clinical evidence indicating benignancy would be extremely important to avoid unnecessary procedure and damage to the patients.

Herein, we reported a hemorrhagic cholecystitis case where we were unable to eliminate the differential diagnosis of gallbladder cancer preoperatively owing to the neglected medication history of long term oral nonsteroidal anti-inflammatory drugs (NSIADs), indicating the importance of reviewing medical history extensively as the standard of preoperative procedure.

## Case presentation

**Fig. 1:**
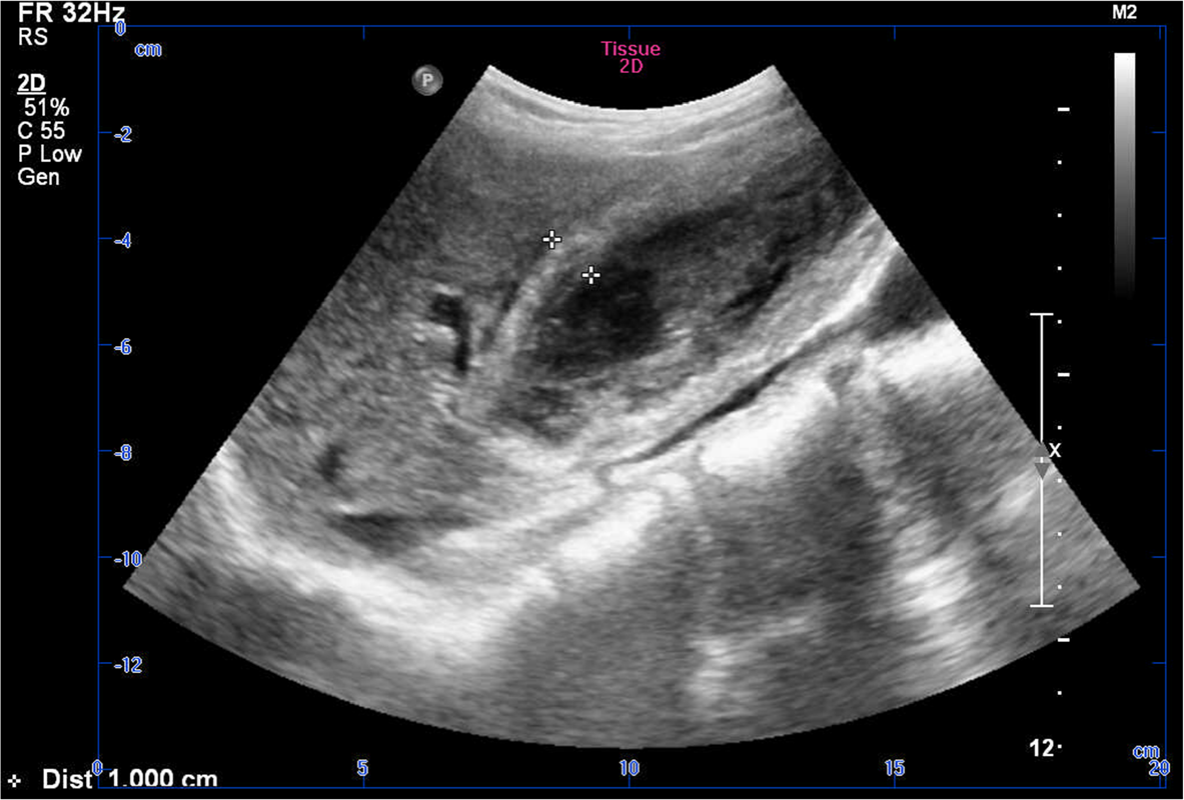
Preoperative ultrasound result indicating the thickened gallbladder wall and hyperechoic structure inside the gallbladder

**Fig. 2:**
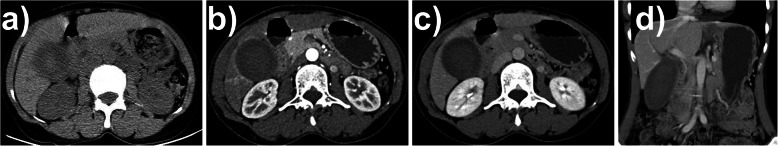
Preoperative CT investigation with **a** non-enhanced, **b** arterial phase, and **c** venous phase transversal images, and **d** coronal view

**Fig. 3:**
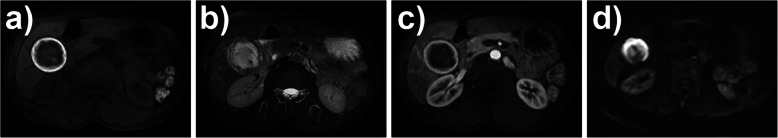
Preoperative MRI evaluation with **a** T1-weighted, **b** T2-weighted, **c** Gd-DTPA contrasted, and **d** DWI images

A 57-year-old Chinese female patient was admitted with slight right-upper-quadrant pain for 2 months, and she did not recall any episode of severe pain. She had a long history of headache but had not visited any neurologist. She was afebrile, without positive Murphy’s sign or any other acute syndrome. Lab tests showed normal complete blood count, coagulation, tumor marker, and liver function results. Specifically, the levels of CA19–9, CA125, and CEA were 21.83 U/mL (normal reference range < 27 U/mL), 9.11 U/mL (normal < 35 U/mL), and 1.28 ng/mL (normal < 5 ng/mL), respectively. Initial ultrasound evaluation indicated hyperechoic structure inside the gallbladder and thickened gallbladder wall (Fig. 1). An enhanced CT scan showed irregular presentations of an enlarged gallbladder (10.0 cm × 4.6 cm × 4.3 cm), with a rough and thickened gallbladder wall and median-density lesions inside the gallbladder (Fig. 2). Notably, there was also a fusion of multiple lesions forming a thick, high-density layer attached to the inner wall. Further MRI showed a high T1-weighted and a low T2-weighted smooth-edged signal on the inner surface (Fig. 3). A flocculent lesion with mixed-density signal was confirmed by MRI. Further gadopentetate dimeglumine (Gd-DTPA) contrasted scan showed signal enhancement of the gallbladder wall, and diffusion weighted imaging (DWI) analysis demonstrated a significantly high signal with smooth edge inside the gallbladder.

**Fig. 4:**
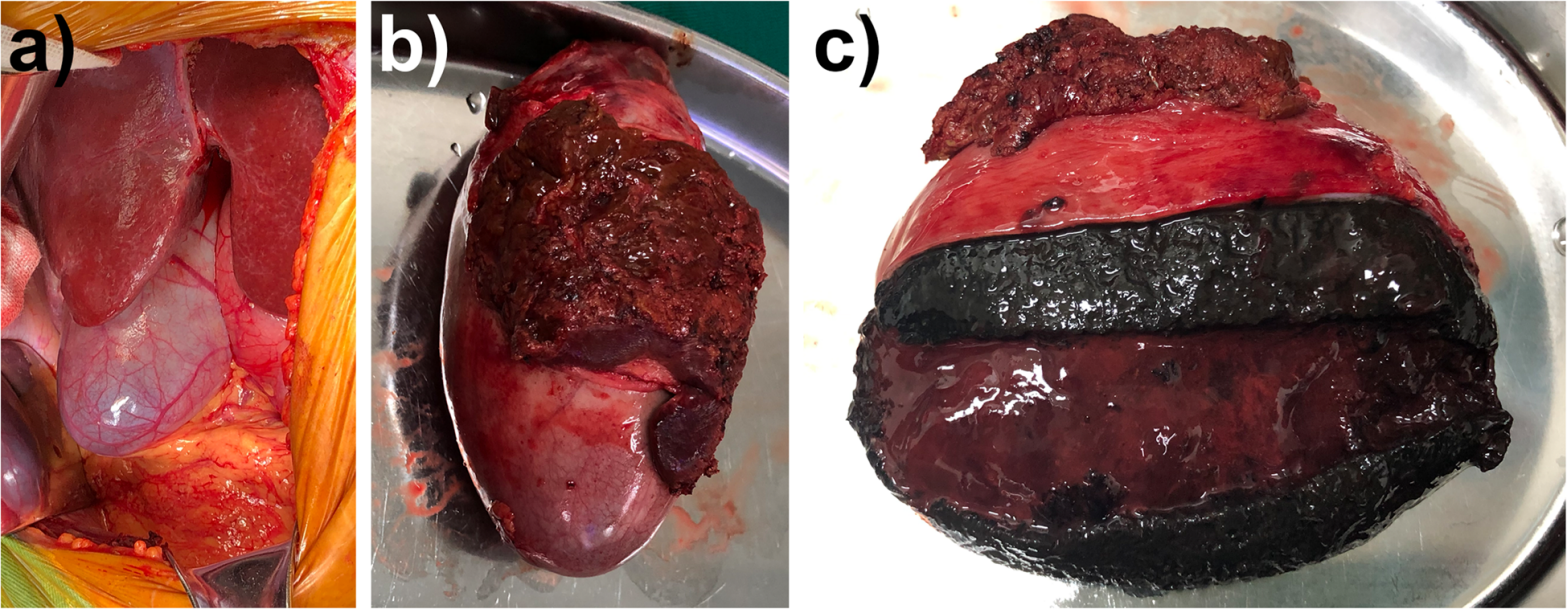
Gross dissection of the gallbladder. **a** The operation field before resection. **b** The resected gallbladder with adjacent liver tissues. **c** The dissected gallbladder with layered content of a slightly rough gallbladder wall, a solid mixture of clot and gallbladder sludge, and a central jelly-like clot

The patient denied further PET-CT examination. The multidisciplinary board could not rule out the possibility of gallbladder malignancy based on the current clinical data, especially considering the rare and atypical imaging presentations. Therefore, an open en-bloc resection of gallbladder and partial liver segments IVb and V were performed to completely remove the suspected lesions. The gallbladder appeared to have high tension and smooth surface. The gross dissection of the enlarged gallbladder showed an interesting presentation inside. From the outside to inside layer by layer, there was a slightly rough gallbladder wall, a solid mixture of clot and gallbladder sludge, and a central jelly-like clot (Fig. 4). Even though a careful inspection and further pathological examination of the gallbladder wall did not reveal any sign of active bleeding, the final pathological result was hemorrhagic cholecystitis, but not gallbladder cancer.

Additional postoperative investigation on the cause of hemorrhagic cholecystitis revealed a neglected medication history. The patient had been frequently using headache powders for the past 6 years to control headache by herself without having melena. The headache powder she used was an over-the-counter (OTC) pain reliever with the brand name “A Ga Fen San”. Each dose contains 230 mg aspirin, 125 mg paracetamol, and 30 mg caffeine - mainly NSAIDs that could significantly increase the risk of bleeding. The patient stated that “A Ga Fen San” could be easily purchased online without any prescription. No medical advice or caution note had been given to her before or during the oral administration of this drug. She was not aware that “A Ga Fen San” should be counted into the current medications list, due to the fact that this brand name is similar to some herbal health products.

The patient recovered well from surgery with no postoperative complication, and was referred to a neurologist for treatment of headache. We advised her to cease self-administrated NSAIDs.

## Discussion and conclusions

This case features a hemorrhagic cholecystitis patient whose radiological presentation showed a distinguishable lesion uniformly distributed on the inner surface of the gallbladder wall. The unique imaging characteristics of blood clot and its mixture with gallbladder sludge increased the difficulty to establish the correct diagnosis. All the routine imagining approaches might help establish the diagnosis of hemorrhagic cholecystitis, but also have their own limitations. Ultrasonography, the initial approach in common setting, could help evaluate the diagnosis, yet the atypical appearance demonstrated in the ultrasonographic studies may lower the diagnostic accuracy by unexperienced ultrasonographers [9]. CT findings like increase bile density within the gallbladder may indicate hemorrhagic cholecystitis, which also requires experienced radiologists to raise the suspicion of hemorrhage [10]. MRI could be applied to non-emergency situations featuring smooth-edged lesion with a high or mixed signal intensity on T1-weighted images, due to methemoglobin resulted from onset of intra-gallbladder bleeding [11]. The characteristic signal remains high or mixed intensity in contrasted scan, which might help to rule out the possibility of gallbladder malignancy incidence and to establish the diagnosis of hemorrhagic cholecystitis.

Multiple risk factors could induce hemorrhagic cholecystitis, such as anticoagulation, blunt trauma, and spontaneous hemorrhage in malignant or bleeding diathesis [12, 13]. In particular, gastrointestinal malignancies, such as gallbladder cancer and metastatic cancer, might cause hemorrhagic cholecystitis [14–16], yet the incidence of such complication remains rare in large clinical trials for advanced gallbladder cancer [17, 18].

In our case, the neglected long-term history of NSIADs medications was the major contributing factor resulting in hemorrhagic cholecystitis. The challenge to diagnose for hemorrhagic cholecystitis is further enhanced due to the confused clinical presentation with other gallbladder diseases such as acute calculous cholecystitis. Several case reports have shown that NSAIDs, particularly antiplatelet agents like aspirin, is a cause of hemorrhagic cholecystitis [19–23], but there is no epidemiological report yet (Table 1). The NSAIDs caused the inhibition of platelet function, which might increase the bleeding risk of the gallbladder lumen when erosive injuries by gallbladder stone were presented. Severe hemorrhagic cholecystitis could also induce hypovolemic shock, which is associated with high mortality rate and requires emergent resuscitation [24]. Fortunately, the patient in current case did not suffer any acute life-threatening bleeding.

**Table 1:** Summary of hemorrhagic cholecystitis cases with NSAIDs use

Age (years)	Gender	Anticoagulation regimen	Reason for anticoagulation regimen	Duration before hemorrhagic cholecystitis	Ref
85	Female	Aspirin 81 mg & warfarin 2 mg QD	Status post sigmoid colectomy	10 days	[19]
75	Female	Aspirin & heparin (dose unknown)	Unstable angina	3 days	[20]
91	Female	Aspirin 325 mg QD & cilostazol (dose unknown)	Lower extremity claudication & cardiovascular protection	Aspirin for 9 years, cilostazol for 4 years	[21]
74	Female	Aspirin 75 mg QD	Myocardial infarction post triple coronary artery bypass	2 months	[23]
51	Female	Aspirin 200 mg QD	Cerebral aneurysms post interventional surgery	3 years	[22]

In addition, the case raised the concern that current medication history could be easily ignored by the physicians or unintentionally omitted by the patients or their relatives. Specifically, in this case, there were complicated contributing factors that eventually caused hemorrhagic cholecystitis. First of all, the attending physicians did not work through the current medication list carefully to look for diagnostic evidence when many confounding differential diagnoses existed. The awareness of NSAIDs use would facilitate the establishment of correct diagnosis and minimize the resection extent, which could significantly reduce the length of hospital stay and medical cost. Secondly, the patient was unaware of the self-medication abuse to treat her headache. Without proper medical advice, she did not know the increased risk of gastrointestinal bleeding or perforation due to the long-term administration of oral NSAIDs [25, 26]. Finally, the OTC NSAID medication used by this patient is obtained easily from online stores or community pharmacies without any prescription. The brand name and package resembled many herbal health products, confusing the patients with the false impression that the medication could be safe even in long-term use. A stricter drug control policy should be applied to NSAIDs prescription and distribution to avoid future abuse.

In conclusion, a hemorrhagic cholecystitis patient in this case report received an en-bloc cholecystectomy and partial liver resection for unclear differential diagnosis and suspected malignancies due to the neglected medication history of long-term oral NSAIDs use. Although diagnosing hemorrhagic cholecystitis remains difficult, the clear medication history of NSIADs use would help to rule out the differential diagnosis of gallbladder cancer. Improved patient education and drug regulatory rule on OTC NSAIDs use should be necessary in the future.

## Data Availability

All data generated or analysed are included in this article.

## Abbreviations

CT: Computed tomography
DWI: Diffusion-weighted imaging
Gd-DTPA: Gadolinium-diethylenetriamine pentaacetic acid
MRI: Magnetic resonance imaging
NSAIDs: Nonsteroidal anti-inflammatory drugs
OTC: Over-the-counter
PET-CT: Positron emission tomography–computed tomography
